# The Relationship between Metabolic Syndrome and Osteoporosis: A Review

**DOI:** 10.3390/nu8060347

**Published:** 2016-06-07

**Authors:** Sok Kuan Wong, Kok-Yong Chin, Farihah Hj Suhaimi, Fairus Ahmad, Soelaiman Ima-Nirwana

**Affiliations:** 1 Department of Pharmacology, Faculty of Medicine, Universiti Kebangsaan Malaysia, Jalan Yaacob Latif, Bandar Tun Razak, 56000 Cheras, Kuala Lumpur, Malaysia; jocylnwsk@gmail.com (S.K.W.); chinkokyong@ppukm.ukm.edu.my (K.-Y.C.); 2 Department of Anatomy, Faculty of Medicine, Universiti Kebangsaan Malaysia, Jalan Yaacob Latif, Bandar Tun Razak, 56000 Cheras, Kuala Lumpur, Malaysia; farihah@ppukm.ukm.edu.my (F.H.S.); apai.kie@gmail.com (F.A.)

**Keywords:** bone, dyslipidaemia, hyperglycaemia, hypertension, obesity, osteoporosis

## Abstract

Metabolic syndrome (MetS) and osteoporosis are two major healthcare problems worldwide. Metabolic syndrome is a constellation of medical conditions consisting of central obesity, hyperglycemia, hypertension, and dyslipidemia, in which each acts on bone tissue in different ways. The growing prevalence of MetS and osteoporosis in the population along with the controversial findings on the relationship between both conditions suggest the importance for further investigation and discussion on this topic. This review aims to assess the available evidence on the effects of each component of MetS on bone metabolism from the conventional to the contemporary. Previous studies suggested that the two conditions shared some common underlying pathways, which include regulation of calcium homeostasis, receptor activator of NF-κB ligand (RANKL)/receptor activator of the NF-κB (RANK)/osteoprotegerin (OPG) and Wnt-β-catenin signaling pathways. In conclusion, we suggest that MetS may have a potential role in developing osteoporosis and more studies are necessary to further prove this hypothesis.

## 1. Introduction

Metabolic syndrome (MetS) and osteoporosis are two seemingly unrelated conditions, yet previous studies have demonstrated the relationship between these two conditions with inconsistent and contradictory findings. A joint interim statement proposed MetS as a multiplex of conditions include abdominal obesity, dyslipidemia (high triglyceride and low high-density lipoprotein (HDL) cholesterol levels), hyperglycemia, and hypertension, which predispose individuals to heart disease, stroke, and diabetes. An individual with at least three of these conditions is considered to have MetS [1]. The prevalence of MetS varies from 10% to 84%, depending on the gender, age, race, and ethnicity [2]. Approximately a quarter of the adult population, worldwide, has MetS, making it a significant public health challenge [3]. The prevalence of MetS in Asia is also increasing to a level similar to the Western countries [4,5].

Osteoporosis is characterized by skeletal fragility and susceptibility to fracture attributed to reduction of bone mass and deterioration of bone micro-architecture [6]. It is a metabolic bone disease occurring in both men and women, particularly when they grow older. Osteoporosis is a major health problem due to the high morbidity, mortality, and significant health care cost involved. Osteoporosis affects more than 200 million people globally [7]. Osteoporosis causes 1.3 million fractures, with 500,000 vertebral, 250,000 hip, and 240,000 wrist fractures costing $10 billion per annum in the USA [8].

The accretion in the prevalence for both diseases prompts the need to understand the relationship between MetS and osteoporosis. The occurrence of both diseases is basically dependent on lifestyle, genetic, metabolic, nutritional, and hormonal factors [9]. Abdominal obesity, dyslipidemia, hyperglycemia, and hypertension are factors associated with the occurrence of osteoporosis, which are also components of MetS [10]. Each feature might give different impacts on bone health. Abdominal adiposity is associated with osteopenia and osteoporosis [11,12]. However obesity, overweight, and high BMI protect against bone loss and subsequently decrease fracture risk [13,14]. In addition, previous studies reported both positive and negative association between dyslipidemia and osteoporosis [15,16]. Additionally, there are contradictory reports on the effects of hyperglycemia [17] and hypertension [18,19] on bone health. The understanding of underlying cellular mechanisms linking both MetS and osteoporosis is necessary to determine whether MetS is positively or negatively related to the risk of bone fractures. We believe that both positive and negative influences of MetS on bone exist in parallel. However, we are not sure whether the net outcome is positive or negative. In this review, evidence of bone loss in animal models of MetS was presented, followed by evidence from human epidemiological studies. The pathogenesis of bone loss due to MetS as evidenced by *in vitro* studies was also discussed. We hope this review provides the readers with a clear picture on the relationship between MetS and osteoporosis.

## 2. The Association between MetS and Osteoporosis

### 2.1. In Vivo Studies

Several animal models of diet-induced MetS have been reported using fructose [20,21,22], sucrose [21,23], high-fat [24], high-fructose high-fat [25,26], and high-sucrose high-fat diet [27]. The Nile grass rat, *Arvicanthis niloticus*, was introduced by Noda *et al.* [28] as a novel model of MetS. Nile grass rats spontaneously developed dyslipidemia, hyperglycemia, abdominal fat accumulation, hypertension, and hyperinsulinemia. An *in vivo* study performed using an animal model fed with a Westernized diet (containing high sucrose, lipid, fatty acids, cholesterol, sodium and chloride content; but low proportion of polyunsaturated fatty acid, vitamins, and protein) indicated reductions of bone mineral density (BMD) and bone mineral content (BMC) at the whole body, femoral, and vertebral levels [29]. In a more recent study performed by Li *et al.* [30], MetS was induced using high-fat diet for 16 weeks in C57Bl/6 mice. Findings from this study showed MetS (increased body weight, plasma lipids, insulin, and insulin resistance) in mice induced with alveolar bone loss, osteoclastogenesis, and inflammation. The current available documented evidence on MetS and osteoporosis in animal studies is still inadequate. Therefore, we need to examine the investigation on the relationship between features of MetS and bone loss in animal studies as well.

#### 2.1.1. Obesity

Previous studies by Halade *et al.* [31] introduced a simple and convenient method in the establishment of novel model for age-associated postmenopausal osteoporosis with obesity induced by high-fat (HF) diet containing 10% corn oil using 12-month old female C57Bl/6J mice. The high-fat diet was given to the mice for six months raised body weight, visceral fat mass, abdominal fat mass, fasting serum glucose, fasting serum insulin, while BMD was reduced. Continuous oral administration of HF corn oil diet for half a year induced an accumulation of adipocytes in mice. Another study utilizing HF diet to induce obesity in male C57Bl/6J mice found reduced BMD, trabecular and cortical bone densities at the tibia of obese mice. Apart from that, bone histomorphometric analysis showed infiltration of adipocytes in the bone marrow of obese mice [32]. The most recent study demonstrated that obese mice fed with a high-fat diet had decreased trabecular bone volume and cortical bone growth shown by micro-computed tomography analysis [33].

#### 2.1.2. Dyslipidemia

Obesity and dyslipidemia often co-exist due to accumulation of visceral fat. Graham *et al.* [34] reported that HF diet was able to cause hypercholesterolemia in mice, evidenced by high total cholesterol, LDL, and unesterified cholesterol. These abnormalities further affected bone quality as the BMC and trabecular structural parameters in femur and tibia were reduced. In a subsequent experiment done by Pirih *et al.* [35], high-fat diet-induced hyperlipidemia mice demonstrated elevated total serum cholesterol, increased glucose levels, and reduced HDL. Micro-computed tomographic analysis indicated reduction in bone surface and bone volume, suggesting the occurrence of impaired bone remodeling in hyperlipidemia mice. Biochemical analysis of mice serum in hyperlipidemia mice displayed augmented levels of parathyroid hormone (PTH), tumor necrosis factor-α (TNF-α), C-terminal telopeptide of type-1 collagen (CTX; a bone resorption marker), calcium, and phosphorus. At the same time, lower levels of amino-terminal propeptide of type-1 collagen (PINP; a bone formation marker) was found. Another experiment was done to access BMD and bone mechanical strength in hypercholesterolemia mice model induced by high-fat high-cholesterol (HFHC) and sodium cholate diet. Hypercholesterolemic mice showed cortical and trabecular bone loss in the femur and vertebrae, whereby the mechanical strength of the bones decreased significantly [36].

#### 2.1.3. Hyperglycemia

Hyperglycemia is a characteristic of diabetes mellitus, a metabolic disease due to impaired insulin secretion (type I diabetes), insulin action (type II diabetes), or both. Previous studies demonstrated greater glomerular filtration rate, increased urinary calcium, reduced fractional calcium reabsorption, and a 53% reduction in the level of osteocalcin (a marker for bone formation) in streptozotocin (STZ)-induced diabetic rats compared to normal rats [37]. In another investigation done by Amir *et al.* [38], BMD of distal femur and vertebra was significantly reduced in diabetic Cohen rats (non-obese rats of type II diabetes) compared to normal rats.

#### 2.1.4. Hypertension

The significance of calcium metabolism in hypertension and osteoporosis has been reported. In an *in vivo* experiment using spontaneous hypertensive rats (SHR) and normotensive Wistar-Kyoto rats (WKY), SHR had lower BMD and bone magnesium content. Administration of a calcium diet augmented BMD and bone calcium content, while bone magnesium content was reduced in both SHR and WKY. Furthermore, the presence of a low percentage of trabecular bone area and newly-formed bone area in SHR compared to WKY were evaluated by Bastos *et al.* [39]. These findings implied a significant relationship between high blood pressure and bone loss.

Summary of the findings for *in vivo* studies are listed in Table 1. These findings demonstrated that obesity, dyslipidemia, hyperglycemia, and hypertension contributed to the development of osteoporosis in experimental animal models.

### 2.2. Human Studies

In contrast to animal studies, human epidemiological studies yield inconclusive results on the relationship between bone health and MetS, whereby positive, negative, and not significant relationships between the two conditions have been reported. In the Rancho Bernardo Study on 420 men (mean age = 74.2 ± 9.7) and 676 women (mean age = 74.4 ± 10.9), 23.5% of men and 18.2% of women had MetS. The incidence for osteoporotic non-vertebral fractures was significantly higher in women with MetS (OR = 3.76, 95% CI 1.27–11.13), but not in men. Men and women with MetS had lower BMD at total hip relative to those without MetS, implying that MetS may be another risk factor for osteoporotic fracture [14]. A later cohort study displayed a negative relationship between MetS and type II diabetes and BMD in 3458 non-diabetic and 735 diabetic male veterans aged 50 to 76 years. Diabetic subjects had lower hip BMD compared to non-diabetic subjects. The BMD level of diabetic subjects and MetS subjects was similar. The findings suggested both MetS and diabetes are associated independently with lower BMD and higher osteoporosis prevalence in men [48]. Cross-sectional studies have been performed in the Korean population by Kim *et al.* [49] and Hwang and Choi [50]. Study by Kim *et al.* [49] involved a total of 2888 Koreans (1780 elderly men aged 40 years and 1108 postmenopausal women) and MetS subjects were found to have lower BMD at the femoral neck compared to normal subjects. Waist circumference was reported as the most significant negative predictor of BMD among all the MetS components. The study by Hwang and Choi [50] recruited 2548 women subjects aged 18 years and above. They found that women with MetS had a lower vertebral BMD. An increase in the components of MetS presented in the subjects was associated with a lower vertebral BMD. In a recent study conducted by Wang *et al.* [51] involving 9930 Chinese adults aged 40 years or older in China, it was observed that the occurrence of osteoporotic fracture was higher among women with MetS (OR = 1.22; 95% CI 1.12–1.54), but not among men.

A few studies also reported a positive relationship between MetS and bone health. In a retrospective study in the USA involving 8197 subjects aged 20 and above, 22% of the subjects were found to have MetS. Subjects with MetS (0.86 g/cm^2^) were found to have higher femoral neck BMD compared to those without MetS (0.80 g/cm^2^) [52]. There was a significant relationship between muscle mass and MetS and prevalence of osteoporosis among Koreans (1654 men and 1979 women, aged 50–93 years). Body composition and BMD were determined using dual-energy X-ray absorptiometry (DEXA) and findings showed that higher muscle mass and MetS were associated with a lower prevalence of osteoporosis in men and women (OR 0.65, 95% CI 0.46–0.91) [53].

Apart from that, the discrepancies on the effects of each component of MetS and bone health in human studies have also been well-studied (Table 2). Albeit the association between MetS and osteoporosis were well-documented, several limitations need to be addressed in these human studies. Firstly, these cohort studies suffered from selection bias as the subjects were selected from restricted population, thus, generalization of the findings to other populations would be difficult. Secondly, assessment of BMD was only performed at a certain site of the bone, but the relationship of MetS and BMD might differ at other sites. Thirdly, causality between MetS and low BMD could not be evaluated owing to the cross-sectional nature of the study. A longitudinal study is more appropriate for this purpose. Therefore, further comprehensive investigation is required to resolve the contradictions.

### 2.3. Potential Mechanisms Involved in Osteoporosis Due to Components of MetS

Obesity is often related with high BMI, body weight, body fat mass, and visceral fat accumulation. Obesity is traditionally perceived to have protective effects on bone. This is attributed to increased mechanical loading on the skeleton and the ability of adipocytes to converts androgens to 17β-estradiol, which increases BMD [10,64]. Obese individuals had higher fat mass, thus, a larger loading to be borne by the skeleton [65]. However, body fat mass only contributes ~16% and ~25% of total body weight in normal men and women, respectively, while the remaining body composition is lean mass [57]. The relative contribution of fat mass and lean mass to BMD is yet to be resolved. Additionally, adipose tissue is the main source for biosynthesis of estrogen in obese individuals [66]. 17β-estradiol is the most potent estrogen followed by estrone and estriol [67,68]. Prestwood *et al.* [69] reported treatment of low-dose estrogen (17β-estradiol) increased BMD of hip, spine, wrist, and decreased markers of turnover in elderly women aged 65 years and above.

However, later investigations revealed paradoxical outcome whereby excessive fat mass and visceral fat accumulation resulting from obesity led to increased risk of osteoporosis. Adipocytes and osteoblasts are derived from a common progenitor, namely multipotential mesenchymal stem cell in the bone marrow, having an equal propensity for adipocytes or osteoblasts differentiation [31]. The balanced profile of adipocytes and osteoblasts differentiation is modulated by various signaling pathways. Obesity is a result of an abnormal growth of adipose tissue through hypertrophy (increase in cell size) and hyperplasia (increase in cell number) in the body [70]. Adipose tissue functions as energy storage as well as a source of hormones and inflammatory mediators such as tumor necrosis factor-α (TNF-α), interleukin-1β (IL-1β), interleukin-6 (IL-6), C-reactive protein (CRP), leptin and adiponectin [71]. High levels of pro-inflammatory cytokines stimulate osteoclast differentiation and bone resorption through activation of receptor activator of NF-κB ligand (RANKL)/receptor activator of NF-κB (RANK)/osteoprotegerin (OPG) pathway [72,73]. Elevation of leptin production and/or reduced secretion of adiponectin by adipocytes may contribute the transportation of macrophage to adipose tissue resulting in infiltration/accumulation of macrophage in adipose tissue [74]. Macrophages, another source of pro-inflammatory factors, further contributing to the detrimental effects of pro-inflammatory cytokines on bone metabolism in obesity [64]. Thus, chronic low-grade inflammation is a hallmark of obesity, characterized by overproduction of various inflammatory markers in the systemic circulation [71].

**Figure 1:**
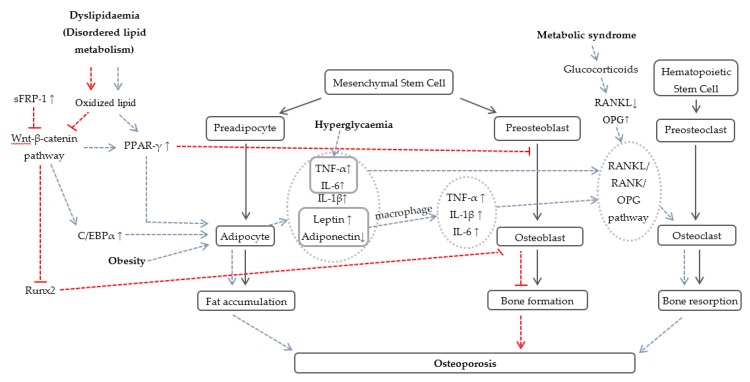
Mechanism of bone loss attributed to increased inflammatory response.

The association between dyslipidemia and osteoporosis is almost similar to obesity because adipose tissue acts as a common feature for both conditions. Disorders of lipid metabolism may lead to high levels of oxidized lipids. Oxidization of lipids stimulates adipocyte differentiation while suppressing osteoblast differentiation through upregulation of peroxisome proliferator-activated receptor-γ (PPAR-γ). This is a member of the nuclear hormone receptor subfamily of transcription factors being expressed in adipocytes to enhance and mediate the differentiation and proliferation of adipocytes [75]. It has also been established that PPAR-γ is a specialized receptor for the detection of fatty acid derived signal molecules, implying its sensitivity towards fatty acids to control lipid metabolism and inflammation. Transient activation of PPAR-γ transduces lipid-mediated inflammatory signaling events [76] (Figure 1). For instance, activated PPAR-γ was reported to enhance adipocyte differentiation and inhibit osteoblast formation in various mesenchymal cell lines and bone marrow [77].

Another plausible mechanism proposed for bone metabolism is the switch between adipocytes and osteoblasts proliferation governed by RANKL/RANK/OPG and Wnt-β-catenin signaling pathway, coordinated by a series of cascade events and transcription factors [78] (Figure 1). Osteoblasts or activated T cells secreted RANKL. RANK is a surface molecule expressed on plasma membrane of osteoclasts. The interaction between RANKL and RANK is a key mediator for osteoclastogenesis and negatively regulated by OPG [79]. OPG (a receptor decoy) is a protein which belongs to the TNF receptor family synthesized by osteoblasts, capable of combining with RANKL, thus inhibiting RANKL-RANK interaction and osteoclastogenesis [80]. A comprehensive *in vitro* and *in vivo* study by Xu *et al.* [32] pinpointed that adipocytes reduced the OPG/RANKL ratio and increased expression of RANK on osteoclasts. Another study illustrated that osteoblasts stimulated with adipocyte-secreted factors increased the expression of RANKL and decreased the production of OPG [31].

The canonical Wnt-β-catenin pathway modulates numerous processes of cell biology, such as the mutually-constrained process between adipocyte and osteoblast differentiation. This pathway determines mesenchymal cell fate and its activation favors osteoblastogenesis. Secreted frizzled-related protein 1 (sFRP-1) is an antagonist of Wnt-β-catenin signaling pathway by binding to Wnt ligand. Overexpression of sFRP-1 often related to obesity or fat accumulation inhibiting *in vitro* osteoblast proliferation through Wnt-β-catenin pathway [81]. A study by Kang *et al.* [82] demonstrated that the activation of Wnt-β-catenin down-regulated C/EBPα and PPAR-γ while upregulated Runx2, leading to activation of osteoblastogenesis and suppression of adipogenesis. Thus, it is hypothesized that fat accumulation and obesity increase expression of sFRP-1, which blocks the Wnt-β-catenin signaling pathway, thereby enhancing adipocyte differentiation through elevation of C/EBPα and PPAR-γ while suppressing osteoblast formation via downregulation of Runx2 (Figure 1).

**Figure 2:**
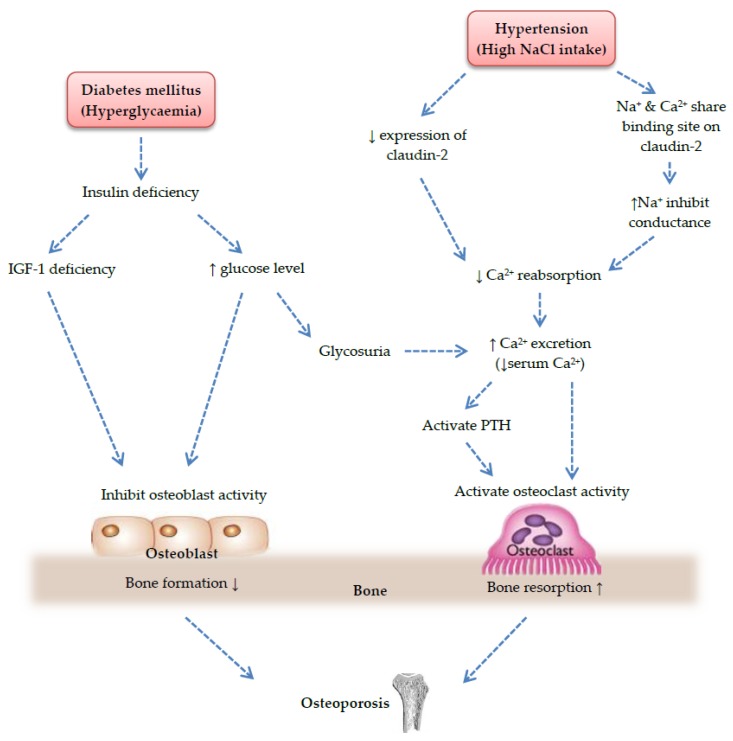
Mechanism of the development of osteoporosis due to disorders of calcium metabolism.

Hyperglycemia is associated with bone loss through the elevation of inflammatory response (Figure 1) and disorders of calcium metabolism (Figure 2). Impaired insulin secretion and/or insulin action emanate from diabetes mellitus increased levels of TNF-α and IL-6. An *in vivo* study using STZ-induced diabetic mice showed that significant elevation in the mRNA expression of TNF-α, macrophage-colony stimulating factor and RANKL caused suppression of bone formation and deterioration of bone strength [83]. On the other hand, *in vitro* experiment suggested that high glucose concentration was responsible to bone loss through changes in bone marrow composition, such as decreased expression of osteocalcin mRNA and Runx2 while promoted PPAR-γ [84]. In diabetes mellitus, the accumulation of advanced glycation end products (AGEs) caused an inferior bone quality and strength. Bone resorption in cultured mouse unfractionated bone cell is enhanced by AGEs. They also induced mesenchymal stem cell apoptosis [85]. Moreover, it was theorized that excessive caloric intakes brought about deposition of fatty molecules in other tissues (muscle and liver) due to overwhelming fat storing capacity of the adipose tissue. Consequently, this phenomenon caused impaired insulin signaling in these tissues, leading to impaired glucose tolerance [86]. Another effect of hyperglycemia on bone turnover is dependent on deficiency of insulin and insulin-like growth factor-1 (IGF-1). IGF-1 is a humoral factor synthesized by liver and osteoblast and acts as a vital anabolic signal to promote bone formation. Deficiency of IGF-1 has been reported to be associated with low BMD, low bone size, growth hindrance, and development of osteoporosis [87,88,89]. An indirect effect of hyperglycemia is glycosuria. According to Schneider *et al.* [90], renal glycosuria caused defective reabsorption of both glucose and calcium in the proximal tubule or collecting duct, leading to hypercalciuria. Therefore, levels of calcium in the body were decreased, thereby affecting bone quality and resulting in bone loss [91].

Abnormality of calcium metabolism is a key factor linking hypertension and osteoporosis (Figure 2). Hypertension is related to high sodium chloride (NaCl) intake, which causes the increase of urinary calcium excretion (hypercalciuria) [92], owing to the competition between sodium and calcium ions in renal proximal tubule [93]. Calcium in blood is filtered by the glomerulus and then reabsorbed into the bloodstream at the proximal distal tubule and ascending loop of Henle in the kidney. The transepithelial permeability of calcium is controlled by proteins in tight junction, claudins [94]. According to a study performed by Muto *et al.* [95], fractional excretion of calcium in knockout mice lacking claudin-2 were greater compared to wild-type mice after administration of NaCl, conjecturing that ingestion of high-salt diet decreased expression of claudin-2 (protein that facilitate calcium reabsorption in proximal tubule) followed by decreased calcium reabsorption and subsequently increased calcium excretion. It has also been reported that sodium and calcium share the common binding site on claudin-2, suggesting that high levels of sodium compete with calcium for binding and conductance [96]. Besides, the reabsorption is often regulated by hormones, such as parathyroid (PTH). Removal of calcium from body through urination decreased circulating calcium level, resulting in the activation of PTH hormone, thus, increased bone turnover [97]. Therefore, it can be postulated that the perpetuation of calcium levels confers protective effects on bone strength and reduced osteoporotic-related fractures in hypertension through curtailment of calcium loss in the urine.

### 2.4. Relationship between Gluco-Mineral-Corticoids, MetS, and Osteoporosis

Another possible factor relating MetS and osteoporosis is the amount of circulating gluco-mineral-corticoids. Effects of glucocorticoid and mineralocorticoid on the development of MetS have been established in human [98,99] and animal studies [100,101]. The profile of endogenous cortisol was reported to positively associate with the rate of bone loss at the lumbar spine, femoral neck, and trochanteric region in elderly healthy men [98]. In another human study, urinary free cortisol and its metabolites were increased in MetS adults with high-sodium intake [99]. A similar outcome was detected in an animal study by Weinstein *et al.* [100]. The results demonstrated endogenous glucocorticoids increased skeletal fragility by reducing spinal BMD in aging C57BL/6 mice. Apart from that, an animal study aimed to assess the involvement of glucocorticoids in obesity induction and triglyceride-rich lipoprotein metabolism, findings displayed adrenalectomy decreased energy intake, body weight, adipose tissue weight, and indexes of triglyceride metabolism in rats fed with a high-sucrose high-fat diet. These effects were reversed by corticosterone replacement, reiterating the potential of glucocorticoids in the development of MetS [101]. Hence, MetS and osteoporosis are the side effect of both endogenous and exogenous glucocorticoids.

## 3. Therapeutic Treatment for MetS and Osteoporosis

Unhealthy eating behavior and sedentary lifestyle are major reasons for the development of MetS. The prevailing clinical management can be divided into lifestyle modification, pharmacologic therapy, and surgery [2,3]. The multi-faceted lifestyle modification may be the safest among all approaches applied for the purpose of losing weight on MetS individuals through a balanced diet, good eating habits, and physical activity. Success in weight reduction may facilitate in reducing abdominal fat mass [102]; decreasing triglycerides while increasing HDL cholesterol levels [103]; lowering fasting blood glucose, insulin, and haemoglobin A1c [104]; as well as lowering blood pressure in hypertensive patients [105]. Appetite suppressants (phentermine derivatives and sibutramine) and nutrient absorption inhibitor (orlistat) are examples of pharmacological approaches for the management of weight loss through reduction of appetite and prevention of fat absorption, respectively [2]. Bariatric surgery, commonly performed through Roux-en-Y Gastric Bypass (RYGB), Sleeve Gastrectomy (SG), or Adjustable Gastric Band (AGB), is only applicable for MetS patients with severe obesity whereby diet modifications, physical activities, and medications do not provide promising effects. Sjöström *et al.* [106] reported the recovery from diabetes, hyperuricemia hypertriglyceridemia, low levels HDL cholesterol, and hypertension in post-surgery patients through gastric bypass, vertical banded gastroplasty, or banding.

Regimen for osteoporosis is also inclusive of lifestyle alterations and pharmaceutical therapy [107]. Therapeutic management strategies in terms of improving lifestyle measures for the prevention and treatment of osteoporosis include calcium and vitamin D supplementation [79], gluten-free diet [108], high protein intake [109,110], exercise [111,112], and smoking cessation [113]. Current development of drugs available for treatment of osteoporosis fractures includes anti-resorptive (denosumab, biophosphonates, and calcitonin) or anabolic agents (teriparatide, strontium ranelate, raloxifene). Anti-resorptive drugs are incorporated by osteoclast and have greater tendency to bind with bone to suppress bone resorption [79]. For osteoporosis in men, pre- and post-menopausal women, bisphosphonates (alendronate or risedronate) are used as the first-line therapy [114]. Intolerance of oral bisphosphonate can be substituted with intravenous zoledronic acid [115,116]. The promising effect of bisphosphonates towards glucocorticoid-induced osteoporosis has been demonstrated to increase lumbar spine BMD in osteoporotic patients [117]. A drug intervention study revealed the potential of RANKL as a drug target. Subcutaneous administration of denosumab (a human monoclonal antibody against RANKL) prevents RANKL-RANK interaction producing a protective effect on the incidence of fractures in postmenopausal females with osteoporosis [118].

A novel treatment has been established for obesity and osteoporosis by targeting apoptotic pathways of adipocytes. Leptin treatment in leptin-deficient (*ob*/*ob*) mice suppressed food intake, reduced body weight, lowered body fat levels, increased energy expenditure, induced adipocyte apoptosis, and increased BMD and bone formation [119]. Additionally, lipid-lowering treatment reduced osteoporosis. Statins, a lipid-lowering medication helps to reduce serum cholesterol, has been reported to increase BMD in postmenopausal women [120]. Several criteria also have to be taken into consideration for management of osteoporosis in diabetic patient. Examples include avoidance of thiazolidenediones (TZDs), exerting good glycemic control, as well as taking calcium and vitamin D supplementation [91]. Researchers have also reported denosumab (a FDA-approved osteoporotic drug) increased human pancreatic beta cells proliferation [121]. Meanwhile, animal studies showed that treatment of hypertensive mice with an angiotensin-converting enzyme (ACE) inhibitor (e.g., enalapril) improved osteoporosis and hypertension [122,123]. Therefore, in our opinion, the curtailment of MetS progression may help in preventing bone loss. However, there are still no drugs available that can halt both diseases.

## 4. Summary and Outlook

The understanding of the association between MetS and osteoporosis must be prefaced with the development of therapeutic intervention on both diseases concurrently. Here, we propose that disturbed calcium homeostasis, induction of inflammatory response, and oxidative stress are common criteria that link components of MetS, as well as the coupling process of bone formation and bone resorption. The existing evidence suggests that correction of MetS should be taken into consideration in the prevention of osteoporotic fracture. It is noteworthy that no single treatment is known hitherto for ameliorating these conjoint diseases. Further studies should be conducted to establish an appropriate *in vivo* animal model mimicking the development of diet-induced MetS. Subsequently, new agents can be developed to treat the conditions associated with MetS and prevent the complication of osteoporosis. Additionally, the influence of sex, age, race, ethnicity, lifestyle, and eating habits should be taken into consideration in investigating the relationship between MetS and osteoporosis in humans.

## Figures and Tables

**Table 1:** Effects of each component of MetS on bone health in *in vivo* experiments.

Researcher (Year)	Types of Animal Model	Findings
**Obesity**		
Nunez *et al.* 2007 [40]	Calorically dense diet-induced obese ovariectomized mice	↑ body adiposity, ↑ leptin; ↓ BMD, ↓ BMC
Halade *et al.* 2010 [31]	HF diet-induced obese mice	↑ body weight, ↑ total body fat mass, ↑ abdominal fat mass; ↓ BMD
Xu *et al.* 2013 [32]	HF diet-induced obese mice	↓ tibia weight, ↓ BMD of tibia, fat cells accumulated in bone marrow of obese mice
Fujita & Maki 2015 [33]	HF diet-induced obese mice	↑ body weight, ↑ total cholesterol, ↑ DL cholesterol, ↑ leptin, ↑triglyceride, ↑ Tb.Sp; ↓ BV/TV, ↓ Tb.N, ↓ Tb.Th
**Dyslipidemia**		
Parhami *et al.* 2001 [41]	Atherogenic HF diet-induced hyperlipidemic mice	↓ femoral mineral content, ↓ femoral mineral density, ↓ vertebral mineral content, ↓ osteocalcin
Graham *et al.* 2010 [34]	HF diet-induced hypercholesterolemic mice	↑ total cholesterol, ↑ LDL, ↑ unesterified cholesterol; ↓ BMC value in femur and tibia, ↓ trabecular bone volume, thickness, and number
Pirih *et al.* 2012 [35]	HF diet-induced hyperlipidemic mice	↓ cortical bone volume fraction (BV/TV), ↑ cortical porosity, ↓ bone strength and stiffness, ↓ PINP; ↑ PTH, ↑ TNF-α, ↑ CTX
Pelton *et al.* 2012 [36]	HFHC diet-induced hypercholesterolemic mice	↑ triglyceride; ↓ BMD, ↓ failure load, ↓ energy to fracture
**Hyperglycemia**		
Ward *et al.* 2001 [37]	STZ-induced diabetic rats	↑ urinary calcium, ↓ bone formation marker
Amir *et al.* 2002 [38]	Cohen diabetic rat	↓ BMD in distal femur and vertebra
Grasemann *et al.* 2012 [42]	Autosomal dominant diabetic mice (hypoinsulinemic hyperglycemia Akita mice)	↓ body weight, impaired glucose tolerance, ↓ whole body BMD, ↓ trabecular bone mass
Liu *et al.* 2013 [43]	STZ-induced diabetic rats	↓ BMD in femur, ↓ numbers of osteoblasts
**Hypertension**		
Metz *et al.* 1990 [44]	SHR	↓ BMD, ↓ bone magnesium
Wang *et al.* 1993 [45]	SHR (26-week-old)	↓ body weight, ↓ BV/TV, ↓ Tb.Th, ↓ Tb.N, ↓ number of osteoblasts and osteoprogenitor cells; ↑ blood pressure, ↑ number of osteoclasts
Wright & DeMoss, 2000 [46]	SHR (24-week-old)	↑ bone turnover in both male and female rats
Bastos *et al.* 2010 [39]	SHR	↓ percentage of trabecular bone area, ↓ percentage of newly-formed bone area
Lee *et al.* 2014 [47]	SHR (20-month-old)	↑ BV/TV, ↑ Tb.N; ↓ Tb.Sp

Abbreviation: BMC = bone mineral content; BMD = bone mineral density; BV/TV = bone volume/total volume; CTX = C-terminal telopeptide of type-1 collagen; LDL = low density lipoprotein; PINP = amino-terminal propeptide of type-1 collagen; PTH = parathyroid hormone; Tb.N = trabecular number; Tb.Sp = trabecular separation; Tb.Th = trabecular thickness; TNF-α = tumor necrosis factor-α.

**Table 2:** Effects of each component of MetS on bone health in human studies.

Researcher (Year)	Types of Study	Findings
**Obesity**		
Edelstein & Barrett-Connor 1993 [54]	Rancho Bernardo Study (1492 ambulatory white adults, 55–84 years)	Body size, waist and hip ratio, BMI, and waist circumference were positively related with high BMD.
Jankowska *et al.* 2001 [12]	Polish men (272 men, 20–60 years)	Visceral adiposity (assessed by waist/hip ratio) contributed to reduced bone mass in men.
De Laet *et al.* 2005 [13]	60,000 men and women from 12 cohorts Rotterdam, EVOS/EPOS, CaMos, Rochester, Sheffield, Dubbo, EPIDOS, OFELY, Kuopio, Hiroshima, and two cohorts from Gothenburg	Low BMI was associated with higher risk for all fractures.
Pesonen *et al.* 2005 [55]	Kuopio Osteoporosis Risk Factor and Prevention Study (1873 women, 48.0–59.6 years)	Premenopausal women had higher BMD, menopausal women had lower BMD.
Yamaguchi *et al.* 2009 [56]	187 men (28–83 years) and 125 postmenopausal women (46–82 years) with type 2 diabetes	Visceral fat (men) and hyperinsulinemia (women) increased FN-BMD in diabetic, protecting against vertebral fracture.
Zhao *et al.* 2007 [57]	Chinese (878 pre-menopausal women, 1110 men; 19.6–45.1 years); Caucasian (2667 females, 1822 males; 19.1–90.1 years)	Increased fat mass did not have a beneficial effect on bone mass.
Greco *et al.* 2010 [58]	398 obese patients (291 women, 107 men; age = 44.1 + 14.2 years)	Obese individuals had low lumbar BMD.
**Dyslipidemia**		
Yamaguchi *et al.* 2002 [16]	214 Japanese postmenopausal women (47–86 years)	High LDL and low HDL cholesterol levels caused low bone mass; high triglycerides levels caused low incidence of vertebral fractures in postmenopausal women.
Adami *et al.* 2004 [15]	2 cohorts: 236 pre- or post-menopausal (35–81 years old); 265 men and 481 women (68–75 years)	The worse the lipid profile (lower HDL cholesterol and higher LDL cholesterol or triglycerides), the higher the bone mass.
**Hyperglycemia**		
Barrett-Connor & Kritz-Silverstein 1996 [17]	Rancho Bernardo Heart and Chronic Disease Study (411 men and 559 women, 50–89 years)	Hyperinsulinemia only increased BMD in women, but not in men.
Schwartz *et al.* 2001 [59]	Osteoporotic Fractures Study (9654 women, ≥65 years)	Diabetic had increased risk of hip, proximal humerus, and foot fractures.
Hanley *et al.* 2003 [19]	Canadian Multicenter Osteoporosis Study (5566 women and 2187 men, ≥50 years)	Type II diabetes was associated with higher BMD in both men and women.
Bonds *et al.* 2006 [60]	Women’s Health Initiative Observational Cohort (93,676 postmenopausal women)	Women with type 2 diabetes were at increased risk for fractures.
Janghorbani *et al.* 2007 [61]	836,941 participants from 16 eligible studies (two case-control studies and 14 cohort studies)	Type 1 and type 2 diabetes increased risk of hip fracture in men and women.
Yaturu *et al.* 2009 [48]	3458 non-diabetic and 735 diabetic male veterans (50–76 years)	Diabetes lowered BMD resulted in increased incidence of hip fractures in men and higher osteoporosis.
**Hypertension**		
Cappuccio *et al.* 1999 [62]	3676 white women (66–91years)	Hypertension increased calcium losses which might contribute to hip fractures.
Hanley *et al.* 2003 [19]	Canadian Multicenter Osteoporosis Study (5566 women and 2187 men, ≥50 years)	Hypertension and type II diabetes were associated with higher BMD in both men and women.
Gotoh *et al.* 2005 [63]	68 non-diabetic women with or without hypertension	Hypertension: ↓ BMD, ↑ calcium/sodium excretion ratio, ↑ PTH, ↑ 1,25(OH)_2_D.

Abbreviation: 1,25(OH)_2_D = 1,25-dihydroxyvitamin D; BMD = bone mineral density; BMI = body mass index; FN-BMD = femoral neck bone mineral density; HDL = high density lipoprotein; LDL = low density lipoprotein; PTH = parathyroid hormone.

## Abbreviations

The following abbreviations are used in this manuscript:

1,25(OH)_2_D	1,25-dihydroxyvitamin D
BMC	bone mineral content
BMD	bone mineral density
BMI	body mass index
BV/TV	bone volume/total volume
C/EBPα	CCAAT-enhancer binding protein α
CTX	C-terminal telopeptide of type-1 collagen
DEXA	dual-energy X-ray absorptiometry
FN-BMD	femoral neck bone mineral density
HDL	high density lipoprotein
HF	high-fat
HFHC	high-fat high-cholesterol
IGF-1	insulin-like growth factor-1
IL-1β	interleukin-1β
IL-6	interleukin-6
LDL	low density lipoprotein
MetS	metabolic syndrome
NaCl	sodium chloride
OPG	osteoprotegerin
PINP	amino-terminal propeptide of type-1 collagen
PPAR-γ	peroxisome proliferator-activated receptor-γ
PTH	parathyroid hormone
RANK	receptor activator of NF-κB
RANKL	receptor activator of NF-κB ligand
Runx2	runt-related transcription factor-2
sFRP-1	secreted frizzled-related protein 1
SHR	spontaneous hypertensive rats
STZ	streptozotocin
Tb.N	trabecular number
Tb.Sp	trabecular separation
Tb.Th	trabecular thickness
TNF-α	tumor necrosis factor-α
WKY	Wistar-Kyoto rats
